# Altered Gene Expression of RNF34 and PACAP Possibly Involved in Mechanism of Exercise-Induced Analgesia for Neuropathic Pain in Rats

**DOI:** 10.3390/ijms18091962

**Published:** 2017-09-13

**Authors:** Shintaro Yamaoka, Yusuke Oshima, Hideki Horiuchi, Tadao Morino, Masayuki Hino, Hiromasa Miura, Tadanori Ogata

**Affiliations:** 1 Department of Bone and Joint Surgery, Ehime University Graduate School of Medicine, Shitsukawa, Toon 791-0295, Ehime, Japan; shinyama@m.ehime-u.ac.jp (S.Y.); morino@m.ehime-u.ac.jp (T.M.); hino@m.ehime-u.ac.jp (M.H.); miura@m.ehime-u.ac.jp (H.M.); ogata@m.ehime-u.ac.jp (T.O.); 2 Translational Research Center, Ehime University Hospital, Shitsukawa, Toon 791-0295, Ehime, Japan; 3 Division of Bio-imaging, Proteo-Science Center, Ehime University, Shitsukawa, Toon 791-0295, Ehime, Japan; 4 Ozu Municipal Hospital, Nishiozu, Ozu 795-8501, Ehime, Japan; horiuchiehime@gmail.com

**Keywords:** neuropathic pain, exercise therapy, SNL model, LMD, RNA sequence, RNF34, PACAP

## Abstract

Despite the availability of several modalities of treatment, including surgery, pharmacological agents, and nerve blocks, neuropathic pain is often unresponsive and sometimes progresses to intractable chronic pain. Although exercise therapy is a candidate for treatment of neuropathic pain, the mechanism underlying its efficacy has not been elucidated. To clarify the molecular mechanism for pain relief induced by exercise, we measured *Rnf34* and *Pacap* mRNA levels in the spinal cord dorsal horn of SNL rats, a model of neuropathic pain. SNL model rats exhibited stable mechanical hyperalgesia for at least 6 weeks. When the rats were forced to exercise on a treadmill, mechanical and thermal hyperalgesia were significantly ameliorated compared with the non-exercise group. Accordingly, gene expression level of *Rnf34* and *Pacap* were also significantly altered in the time course analysis after surgery. These results suggest that exercise therapy possibly involves pain relief in SNL rats by suppressing *Rnf34* and *Pacap* expression in the spinal cord.

## 1. Introduction

Many patients in clinics complain of pain in the trunk or extremities. In the field of spine surgery, there are many causes of neuropathic pain, such as traumatic injury, entrapment and compression syndrome, and neoplastic disease. Neuropathic pain, which results from mechanical compression or degeneration of nervous tissue, is associated with allodynia, hyperalgesia, and continuous spontaneous pain. Accordingly, treatment of pain is one of the most important topics for clinicians in orthopedic surgery and anesthesiology. Several therapeutic modalities, including decompression surgery, pharmacological treatment, and nerve block, have been applied for the treatment of neuropathic pain. An operative treatment for lumber disc herniation has consistent evidence that short-term efficacy of surgery is higher than that of conservative treatment, but the long-term efficacy is not significant in compared to conservative treatment [1], thus surgical treatments are not always effective for neuropathic pain. Regarding those conservative treatments, non-steroidal anti-inflammatory drugs (NSAIDs) are commonly used for the treatment of musculoskeletal pain, but because these drugs act by inhibiting prostaglandin production, they are effective only for treatment of inflammatory pain. Typical pharmacological agents used to treat neuropathic pain include opioid receptor agonists, Ca-channel blockers, and monoamine re-uptake inhibitors. Opioid receptor (OR) and monoamine systems are the primary mechanisms that inhibit transmission of pain sensation. Accordingly, endogenous opioid receptor agonists are very powerful analgesic mechanisms. In the spinal cord, δOR play important roles in antinociception [2]. Clinically, µOR agonists such as morphine are commonly used for the treatment of serious pain, including postoperative pain. Monoamines, such as noradrenalin [3] and serotonin [4], are also strong endogenous pain-relieving agents. Monoamines inhibit pain signals by the activating of γ-aminobutyric acid (GABA) signals [5]. Elevation of monoamines by re-uptake inhibitors has been used for pain relief care in patients with several neuropathic pain [6]. In addition, we reported previously that serotonin reuptake inhibitors ameliorated neuropathic pain induced by spinal cord injury [7]. Pregabalin, a structural analog of GABA that selectively binds the α2-δ (α2-δ) subunit of voltage-dependent calcium channels, possesses analgesic, anxiolytic, and antiepileptic properties. Gabapentin and pregabalin are regarded as first-line treatments for peripheral pain with a neuropathic component [8]. However, pharmacological treatment cannot completely cure all symptoms of neuropathic pain.

Despite the availability of these pharmacological treatments and other conservative treatments such as physical therapy, many patients have intractable chronic neuropathic pain. Actually, to overcome neuropathic pain, we should propose a combination of those treatments. We focused on exercise therapy as an effective candidate of conservative treatment for neuropathic pain. Exercise therapy has been established as the main conservative therapy for patients with chronic lower-back pain [9], and there are few reports indicating that exercise therapy is effective for the treatment of sciatica in human [10]. On the other hand, in animal there are some reports. Stagg et al. reported that exercise training reversed thermal and tactile hypersensitivity in the rat SNL model. In addition, they found that exercise increased β-endorphin and met-enkephalin content in the rostral ventromedial medulla and the mid-brain periaqueductal gray area [11]. The forced exercise training also improved neuropathic pain after spinal cord injury in rats [12]. Leung et al. reported that physical activity alters macrophage phenotype to increase IL-10 and prevent chronic pain in C57BL/6J mice [13]. Bobinski et al. demonstrated that the exercise suppresses pain-like behaviors in animals with neuropathic pain by enhancing brainstem serotonin (5-HT) neurotransmission [14]. In the neuropathic pain rat model, exercise induced analgesia could be mediated by desensitization of central µOR by endogenous opioids [15]. However, recent reports have not fully explained the underlying mechanism by which exercise therapy ameliorates neuropathic pain.

In this study, to clarify the mechanism of the effect of exercise on neuropathic pain due to nerve compression, we subjected SNL rats to enforced exercise on a treadmill and observed the molecular changes in the dorsal horn of the spinal cord.

## 2. Results

First, in order to confirm that ligation of the L5 spinal nerve (SNL model) caused neuropathic pain without deficiency of motor function, we observed hind-limb motor function using the Basso, Beattie, and Bresnahan (BBB) scale (supplementary table). All SNL animals exhibited full hind limb function (BBB score of 21) during the experimental period (data not shown). Pain-like behavior was assessed by mechanical stimulation using the von Frey filament test.

**Figure 1:**
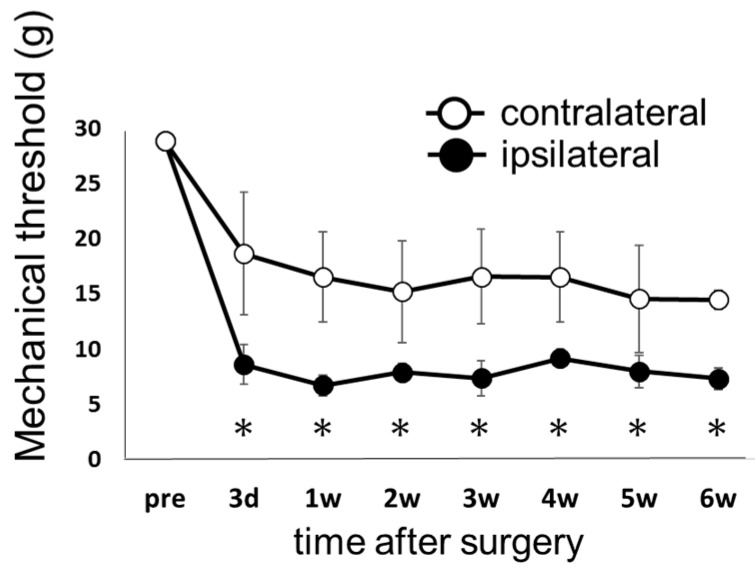
Time course of withdrawal latency in response to mechanical stimulation in the spinal nerve ligation (SNL) model without exercise (the control group). The hind paw withdrawal threshold by mechanical stimulation was determined by von Frey test. Measurements were performed before surgery (pre), and weekly for 6 weeks after surgery (3 days, 1, 2, 3, 4, 5, and 6 weeks). Closed circles (●) represent the side ipsilateral to ligation, and open circles (○) represent the side contralateral to ligation. Data represent averages ± S.D. (*n* = 6), * *p* < 0.05 according to Wilcoxon signed-rank test. Abbreviation: d—days, w—weeks (the same below).

Figure 1 shows the pain threshold in response to mechanical stimulus in the ipsilateral and contralateral hind limbs in the control group. In animals that received SNL, the pain threshold decreased 3 days after surgery on both the ipsilateral and contralateral sides, and reached a plateau that was sustained until 6 weeks after surgery. After the operation, the pain threshold was significantly lower on the ipsilateral side than on the contralateral side. In the sham operation group, no significant change in pain threshold was observed after surgery (data not shown).

**Figure 2:**
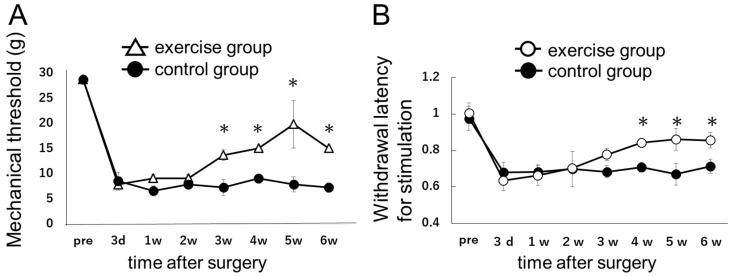
(**A**) Effect of exercise training on mechanical hyperalgesia in the hind paw ipsilateral to ligation in the SNL model. The mechanical threshold in the hind paw ipsilateral to ligation was compared between SNL model rats subjected to (exercise group) or not subjected to (control group) forced treadmill running. Open triangles (Δ) represents the exercise group, and closed circles (●) represents the control group. Data represent averages ± S.D. (*n* = 6), * *p* < 0.05 according to Wilcoxon signed-rank test. Starting 3 weeks after the surgery, exercise training significantly improved neuropathic pain induced by SNL; (**B**) The time course of withdrawal latency for thermal stimulation. Open circles (○) represent the exercise group, and closed circles (●) represent the control group. The withdrawal latencies for thermal stimulation are presented as the ratio of ipsilateral to contralateral. Data represent averages ± S.D. (*n* = 6), * *p* < 0.05 according to Wilcoxon signed-rank test. Starting 3 weeks after the surgery, exercise training gradually improved neuropathic pain induced by SNL. Four weeks after surgery or later, the withdrawal latency of exercise group was larger than that of control group.

Next, we assessed the behavioral effect of exercise on neuropathic pain (Figure 2A). For this purpose, we divided the SNL rats into two groups, exercise and control (i.e., non-exercise).

In the behavior test, the pain threshold decreased 3 days after surgery in both the exercise and the control groups. In the exercise group, pain-like behavior improved starting 3 weeks after surgery. From 3 to 6 weeks after surgery, the pain thresholds were significantly higher (*p* < 0.05) in the exercise group than in the control group.

**Figure 3:**
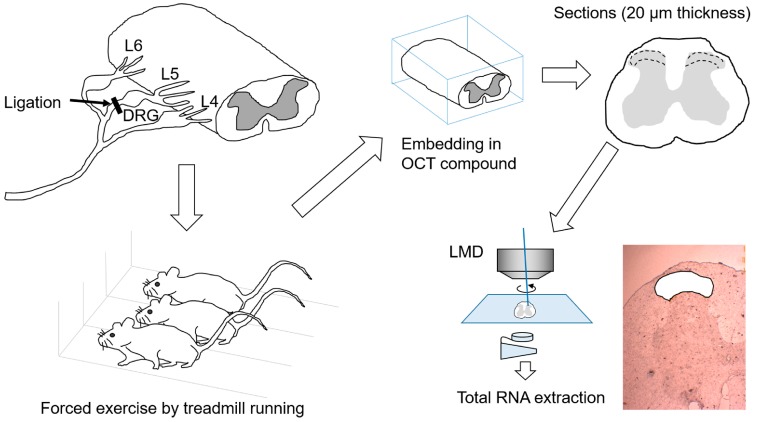
Schematic illustration of experimental procedures. L5 spinal nerve was ligated unilaterally (on the right side) distal to the L5 dorsal root ganglion (DRG). SNL rats were subjected to forced exercise on a treadmill as a model of exercise therapy. Spinal cord was resected at the T11 level and embedded in OTC compound, and then 20-μm frozen sections were produced. The tissue from laminae I–II of the dorsal horn was collected using a laser microdissection method, and the samples were used for extraction of total RNA.

Figure 3 depicts the experimental procedure from the L5 spinal nerve ligation surgery to total RNA extraction.

**Figure 4:**
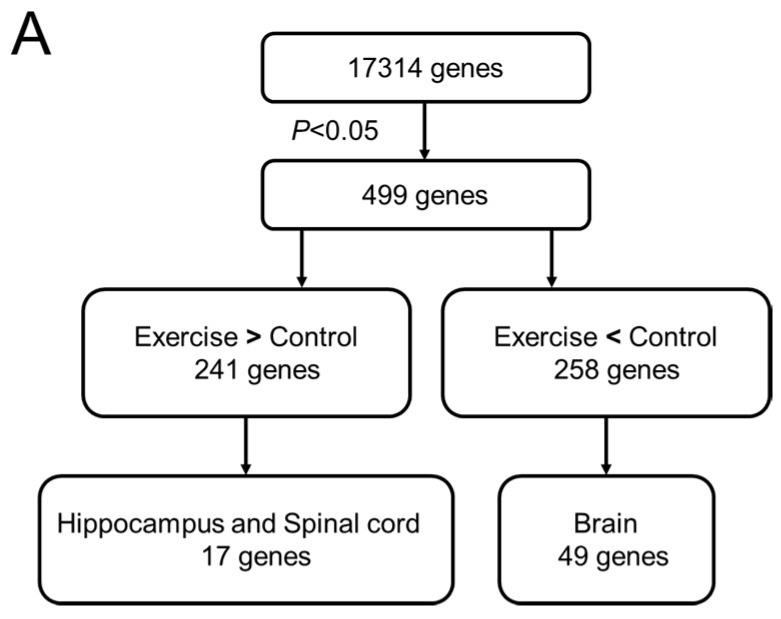

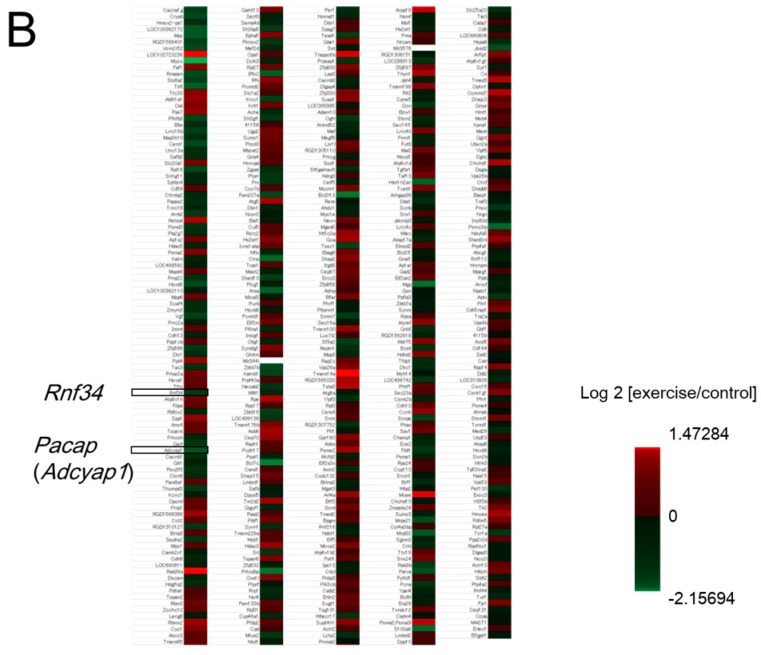
(**A**) Analytical flowchart of gene-expression analyses by RNA-seq, bioinformatics, and quantitative PCR. RNA samples isolated from dissected tissue from the exercise and control (non-exercise) groups 6 weeks after surgery were subjected to RNA-seq. In total, 17,314 genes were initially identified. Those genes were sorted by relative expression level (exercise vs. control). In the exercise group, 241 genes were up-regulated and 258 genes were down-regulated with statistical significance (*p* < 0.05 according to Student’s *t*-test). Those genes were annotated based on the tissue specificity of their expression; (**B**) Heat map of the gene expression profile obtained from the result of RNA-seq. Total of 499 genes (241 upregulated genes and 258 downregulated genes) were identified with statistical significance. *Rnf34* and *Pacap* (*Adcyap1*) were selected from the list of downregulated genes by tissue specific functional annotation. The fold change values (log 2 (exercise/control)) of *Rnf34* and *Pacap* were −0.72 and −1.01, respectively. Clearer picture can be found in the supplementary figure.

To elucidate how exercise therapy ameliorates neuropathic pain, we monitored differential gene expression using a next-generation sequencing (NGS) method, RNA-seq. After the exercise program was complete, we resected the spinal cord tissue and performed gene-expression analyses by RNA-seq and quantitative real-time PCR (RT-PCR), as shown in Figure 4A. RNA-seq yielded about 6,000,000 reads from total RNA samples derived from laminae I–II of the dorsal horn of the spinal cord. Of 17,314 genes identified by mapping onto the rat genome, 499 exhibited significant differences in the expression between the exercise and control groups. Specifically, 241 genes were upregulated in the exercise group, and 258 were downregulated (Figure 4B). Next, we performed tissue-specific functional annotation focusing on the central nerve system, brain, and spinal cord. Of the differentially expressed genes, 17 upregulated and 49 downregulated genes are thought to be mainly expressed in brain and spinal cord (data not shown). Ultimately, we focused on *Rnf34* and *Pacap*, which were significantly downregulated in the exercise group and are thought to be associated with pain.

**Figure 5:**
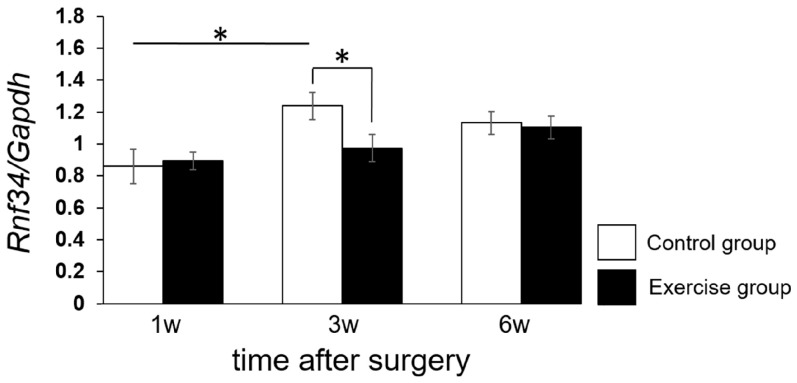
Effect of exercise therapy (treadmill running) on *Rnf34* levels in dorsal horn (laminae I–II). Levels of *Rnf34* mRNA were determined by real-time PCR (RT-PCR). Time course measurements were performed at 1, 3, and 6 weeks after surgery. The results were normalized against the corresponding levels of *Gapdh* mRNA, a housekeeping gene. Values represent means ± S.D. (*n* = 6), * *p* < 0.05 according to Student’s *t*-test and two-way ANOVA, followed by post-hoc Tukey HSD test. The main effect of time dependency yielded an *F* value of *F*(2,30) = 7.3757, *p* = 0.00133.

Next, we performed quantitative RT-PCR on *Rnf34* and *Pacap* and compared their levels between the exercise and control groups at 1, 3, and 6 weeks after surgery. Expression of *Rnf34* was significantly downregulated 3 weeks after surgery in the exercise group, but no significant difference between the exercise group and the control group was detected at 1 or 6 weeks after surgery (Figure 5).

**Figure 6:**
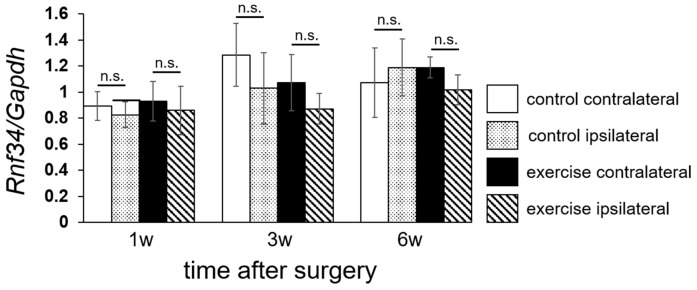
Laterality of *Rnf34* mRNA level in dorsal horn (laminae I–II). Levels of *Rnf34* mRNA, presented in Figure 5, were analyzed separately in the ipsilateral and contralateral sides. Values represent means ± S.D. (*n* = 6). No significant differences (n.s.) were detected by three-way ANOVA. The main effect of laterality yielded an *F* value of *F*(1,60) = 2.471, *p* = 0.121.

To confirm the laterality of *Rnf34* expression level, we separately analyzed the ipsilateral and contralateral sides of laminae I–II, as shown in Figure 6. At 3 weeks after surgery, expression of *Rnf34* was lower on the ipsilateral side than on the contralateral side. A similar tendency was also detectable at 1 and 6 weeks after surgery, but the differences were not significant.

**Figure 7:**
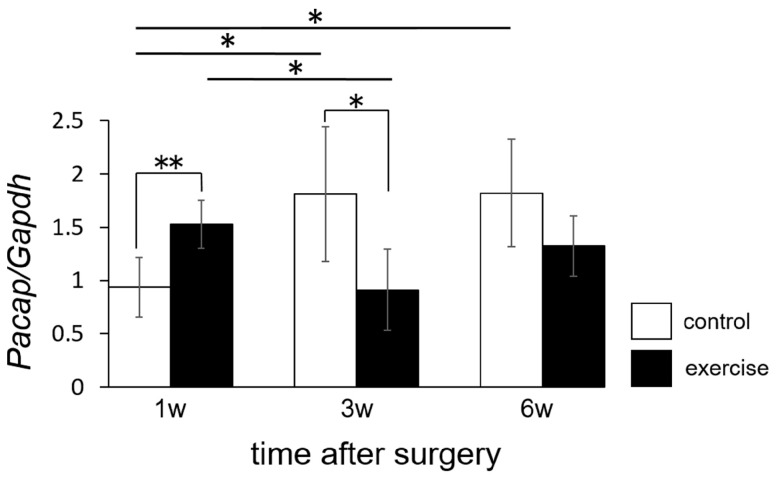
Effect of exercise therapy (treadmill running) on *Pacap* levels in dorsal horn (laminae I–II). Levels of *Pacap* mRNA were determined by RT-PCR. Time course measurements were performed at 1, 3, and 6 weeks after surgery. The results were normalized against the corresponding levels of *Gapdh* mRNA, a housekeeping gene. Values represent means ± S.D. (*n* = 6), * *p* < 0.05, ** *p* < 0.01 according to Student’s *t-*test and two-way ANOVA, followed by post-hoc Tukey HSD test. The interaction effect between exercise and time yielded an *F* value of *F*(2,30) = 8.8204, *p* = 0.001.

We also evaluated expression of *Pacap*. In the exercise group, expression of *Pacap* was significantly upregulated 1 week after surgery, but significantly downregulated 3 weeks after surgery, relative to the control group (Figure 7). Expression of *Pacap* also tended to be downregulated 6 weeks after surgery, but the difference was not significant in comparison to the control group.

**Figure 8:**
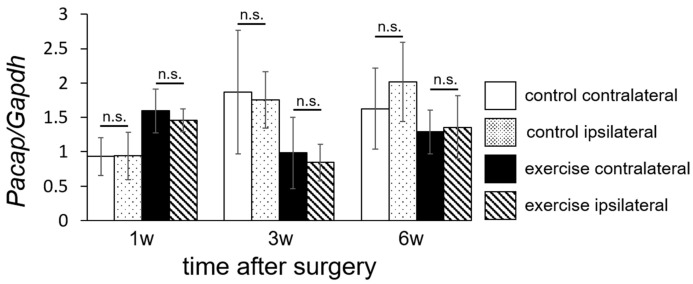
Laterality of *Pacap* mRNA level in dorsal horn (laminae I–II). Levels of *Pacap* mRNA, presented in Figure 5, were analyzed separately in the ipsilateral and contralateral sides. Values represent means ± S.D. (*n* = 6). No significant differences (n.s.) were detected by three-way ANOVA. The main effect of laterality yielded an *F* value of *F*(1,60) = 1.0424, *p* = 0.3114.

There was no significant difference in *Pacap* expression between the ipsilateral and contralateral sides (Figure 8).

## 3. Discussion

In this study, we analyzed molecular changes in the spinal cord dorsal horn in order to clarify the analgesic mechanism of exercise in the treatment of neuropathic pain. We employed the SNL model in this study. This model has been demonstrated to produce neuropathic pain without motor function loss [16]. Consistent with this, all of our SNL animals had maximal BBB score [17], indicating no motor deficiency in the hind limbs. 

We identified two genes, *Rnf34* and *Pacap*, whose expression levels in the dorsal horn were changed by exercise. Ring finger protein 34 (RNF34) is a specific E3 ubiquitin ligase for PGC-1α and one of human ortholog gene family. The protein was initially identified RING finger homologous to IAP type (hRFI) [18,19]. It was recently shown that the ring finger domain has E3 ubiquitin activity that targets caspase-8 and -10 in death receptor–mediated apoptosis [19], and that exogenous overexpression of hRFI in colorectal cancer cells inhibits the extrinsic apoptotic pathway [20]. Ubiquitin (Ub) ligation is implicated in active protein metabolism and subcellular trafficking, and its impairment is involved in various neurologic diseases [21]. Jin et al. reported that RNF34 reduces the expression of the γ2 GABA_A_R subunit by increasing the ratio of ubiquitinated to nonubiquitinated γ2. Overexpression of RNF34 in hippocampal neurons decreases the density of γ2 GABA_A_R clusters and the number of GABAergic contacts received by these neurons. shRNA-mediated knockdown of endogenous *Rnf34* leads to elevated γ2 GABA_A_R cluster density and GABAergic innervation. Jin et al. concluded that RNF34 regulates postsynaptic γ2-GABA_A_R clustering and GABAergic synaptic innervation [22]. Although it has not been reported that inhibition of Rnf34 directly ameliorated pain sensation, activation of GABA_A_ receptor in spinal dorsal horn has been established to be one of the most powerful analgesic mechanisms in mammals. Pharmacologic removal of GABA_A_ receptor-mediated neurotransmission elicited pronounced pain hypersensitivity in intact animals [23]. GABA_A_ receptor agonists have, therefore, been proposed as potent analgesics for pathological pain [24]. Afrazi et al. reported that application of allopregnanolone, a neurosteroid, markedly ameliorated diabetes-induced thermal hyperalgesia in rats via preservation of γ2 subunit of GABA_A_ receptor in lumbar dorsal horn [25]. The working mechanism of this substance was inhibition of GABA_A_ receptor down-regulation. Preservation of GABA_A_ receptors may shift the stimulation-pain response from hypersensitive to hyposensitive in the patients with neuropathic pain. Therefore, inhibition of *Rnf34* induces pain relief via preservation of GABA_A_ receptors. As an alternative hypothesis of the possible mechanism for exercise-induced analgesia, Rnf34 is mainly expressed in oligodendrocytes in the CNS [26]. Therefore, it is possible that exercise improves oligodendrocyte/axonal function [27]. Specific oligodendrocyte injury was recently shown to induce neuropathic pain [28].

In this study, we demonstrated that expression of *Rnf34* in the dorsal horn, an area containing postsynaptic receptor for GABAergic transmission, was inhibited by exercise 3 weeks after the operation (Figure 5). Because the GABAergic system is one of the most powerful endogenous analgesic systems, suppression of RNF34 expression might provide pain relief via inhibition of postsynaptic γ2-GABA_A_R clustering under neuropathic pain conditions. We detected an exercise-induced inhibition of *Rnf34* expression 3 weeks after the operation, in comparison with control animals, although we observed no difference between the two groups at 1 or 6 weeks after the operation. This may be because 1 week of exercise may be too brief to allow expression of analgesic reactions. On the other hand, despite persisted pain relief by exercise, it was continued until 6 weeks after the operation (Figure 3), the *Rnf34* mRNA level had risen nearly to the level in the control group at 6 weeks (Figure 5). Inhibition of *Rnf34* expression around 3 weeks after the operation may have decreased the total amount of RNF34 protein in the dorsal horn, and this lower level of RNF34 protein may have persisted until the end of observation (6 weeks after the operation).

PACAP (pituitary adenylate cyclase-activating polypeptide), a neuropeptide that stimulates adenylate cyclase in rat anterior pituitary cell cultures, was originally isolated from ovine hypothalamic tissues by Miyata et al. [29]. PACAP27 and PACAP38 are members of the VIP/secretin/glucagon family of peptides that have diverse neuro-regulatory effects in sympathoadrenal cell development and function [30]. In human cadavers, PACAP-like immunoreactivity is detectable both in dorsal horn and dorsal root ganglia [31]. Narita et al. reported that PACAP induces hyperalgesia in the mouse spinal cord, and detected PACAP38 immunoreactivity in numerous nerve fibers in the superficial layers of the dorsal horn of the cervical, thoracic, lumbar, and sacral segments. Moreover, intrathecal application of PACAP38 elicits pain-like behavior in mice [32].

Zhang et al. observed *Pacap* mRNA expression in L5 dorsal root ganglion after unilateral adjuvant-induced inflammation in the rat paw [33]. Mabuchi et al. reported that mice lacking the *Pacap* gene (*Pacap*^−/−^) do not exhibit inflammatory pain induced by intra-plantar injection of carrageenan or neuropathic pain induced by L5 spinal nerve transection, although they do retain normal nociceptive responses. Intrathecal administration of NMDA results in mechanical allodynia in wild-type mice, but not in *Pacap*^−/−^ mice [34]. Davis-Taber et al. reported that intrathecal application of PACAP receptor antagonist potently reduces mechanical allodynia in a neuropathic spinal nerve ligation model [35]. These reports indicate that inhibition of *Pacap* expression in dorsal horn may relieve pain in patients or animals with neuropathic pain.

In this study, we demonstrated that the *Pacap* mRNA level in the exercise group was just initially higher than that in the control group at 1 week (Figure 7). In our research protocol, forced exercise started the day after the operation. The animals in the exercise group may have experienced higher stress due to the early initiation of exercise after surgery, causing a protein related to pain mechanisms (PACAP) to be activated. On the other hand, in the exercise group, the *Pacap* mRNA level decreased dramatically between 1 week and 3 weeks after the operation and *Pacap* mRNA levels 3 and 6 weeks after the operation were lower in the exercise group than in the control group. Thus, it may take a rather long time (3 weeks) to achieve pain relief via exercise treatment. Suppression of *Rnf34* (Figure 6) and *Pacap* (Figure 8) were observed not only on the injured (ipsilateral) side, but also on the non-injured (contralateral) side 3 weeks after the operation. Thus, the molecular change we observed in the dorsal horn was not target-specific. It is possible that the initial molecular changes induced by exercise were generated at a more proximal level, e.g., in brain cortex or hypothalamus, and that the analgesic signals subsequently spread in a peripheral direction with no distinction between the injured and non-injured sides. The limitation of this study is that we observed the molecular change only in the spinal cord. The molecular change in the patients/animals with neuropathic pain should occur not only in the spinal cord, but also other central nervous tissue such as brain cortex, hypothalamus, and hippocampus. The molecular change by exercise should also be occur in other nervous tissue than spinal cord. Further experiment was necessary to clear up the effect of *Rnf34* or *Pacap* as working mechanism of exercise therapy. To consider possible correlation with other genes detected on the RNA-seq, further bioinformatics analysis, e.g., pathway analysis and hierarchical clustering analysis should be performed in the current data set or in various experimental conditions. Otherwise, it would be important to explore the changes in target molecules such as GABA_A_ receptor and PACAP receptor to reveal the analgesic mechanism for clinical implementation in the future.

In summary, the current study aimed to investigate the working mechanism of endogenous mediators during exercise in the pathophysiology of neuropathic pain. The results of this study provides important clinical significances. The rehabilitation including muscle exercise is usually hard for the patients with pain. Consequently, clarification of the pain relief mechanisms is valuable to provide convincing and satisfactory explanation to the patients. Our results suggest that pharmacological inhibition of *Rnf34* and *Pacap* can be candidates for the treatment of neuropathic pain. The combination of exercise and pharmacological inhibition was also considered as more potent pain treatment. Our study is potentially transferable to future human studies.

## 4. Materials and Methods

### 4.1. Animals

All experimental procedures were conducted in accordance with a protocol approved by the Ethical Committee for Animal Experiments of Ehime University (#05NU73-2, 02 July 2015). A total of 72 female Wistar rats (Charles River Laboratories, Yokohama, Japan) were purchased at 6 to 8 weeks old, and randomly divided into three groups. L5 spinal nerve injury animals divided to two groups: with (the exercise group) and without (the control group) exercise. In some animals, sham operation was performed (the sham group). All rats were subjected to behavioral tests after surgery. The exercise group underwent treadmill running. All rats were sacrificed to harvest spinal cord tissues for further analyses, as described below.

### 4.2. Surgical Procedures

Rats were anesthetized with 1.5–2% (*v*/*v*) isoflurane in air, and then right L5 spinal nerve ligation was performed as described by Kim and Chung [16]. After shaving of hair and sterilization with iodine/70% ethanol, a midline longitudinal incision was made from the L4 to S1 vertebrae, and the right paraspinal muscle was exposed. The paraspinal muscle was then removed from the level of the L5 spinous process to the sacrum. The transverse process of L6 was exposed, and removed. The L5 spinal nerve was tightly ligated with a piece of 5-0 silk distal to the L5 dorsal root ganglion. After nerve ligation, the wound layer of the dorso-lumbar fascia and skin incision were closed with 5-0 silk thread. Sham operation was performed in the same manner, except that nerve ligation after exposure was omitted.

### 4.3. Evaluation of Motor Function

Motor function was assessed with the Basso, Beattie, and Bresnahan (BBB) scoring scale [17], one of the most widely used methods for evaluating hind-limb motor function in rats and mice, a 21-point scale that ranks no locomotion as 0 points and normal gait as 21 points. BBB scoring was performed by three individuals who were unaware of the treatments that the rats had received. Data reflect the averages of the three observers’ scores.

### 4.4. Evaluation of Pain-Like Behavior

To evaluate mechanical sensitivity of the foot, as determined by foot withdrawal threshold in response to mechanical stimuli, we performed the von Frey test using Semmes Weinstein Monofilaments (A835-14-18, SAKAI Medical, Tokyo, Japan). The rats were placed on a metal mesh floor, and von Frey filaments were applied from underneath the metal mesh floor to the foot. To determine the withdrawal threshold, the stimulus strength was sequentially increased and decreased by up-down method. When the rats felt pain and withdrew their paw, the withdrawal threshold was measured by applying forces 5.5, 8.65, 11.7, 15, and 29 g. Paw sensitivity threshold was defined as the minimum pressure at which immediate withdrawal reflex of the paw was observed more than three times in a row. The measurements were performed before surgery and weekly for 6 weeks after surgery (1, 2, 3, 4, 5, and 6 weeks). Thermal sensibility was assessed by using the Hargreaves’ plantar test apparatus (Ugo Basile, Varese, Italy) as previously described [36,37]. In brief, rats were placed on a 2 mm thick glass floor 30 min before the experiment for habituation. A heat generator with an aperture of 10 mm diameter was focused onto the hind paw plantar surface pointing at both the lateral and the medial paw test sites. Thermal withdrawal latency was taken as mean of three measurements per each hind paw, with 5 min interval between each measurement. The withdrawal latencies were recorded in seconds of each paw.

### 4.5. Treadmill Running

The exercise group was assigned to perform interval training programs on a treadmill (MK-680, Muromachi Kikai, Tokyo, Japan). Initial treadmill conditions were as follows: 10 m/min at 10 degrees inclination for 10 min. Treadmill running started the day after the operation, and was performed 5 days per week. The velocity of the treadmill increased by 1 m/min each day until it reached 20 m/min. During the experimental period, the exercised rats exhibited no significant change of body weight in comparison with sedentary controls.

### 4.6. Laser Micro-Dissection (LMD) for RNA Extraction

Spinal cords from the exercise, control, and sham groups were dissected without fixation, immediately embedded in O.C.T. Compound (Tissue-Tek^®^, Sakura Finetek Japan, Tokyo, Japan), and frozen in dry ice/acetone baths. Frozen sections 20 µm thick were processed, and portions of laminae I–II in dorsal horn were clipped out using a laser microdissection system (Leica LMD7000, Leica Microsystems, Tokyo, Japan). About 10 clipped slices of sections were collected per rat, and these clipped sections were collected separately from the ipsilateral and contralateral sides. The clipped sections were immediately subjected to RNA purification using an RNA isolation kit (NucleoSpin^®^ RNA XS, Clontech, TaKaRa, Shiga, Japan). The flow of the research protocol from operation to mRNA extraction is shown in Figure 3.

### 4.7. RNA Sequence and Functional Annotation Bioinformatics

We selected six samples derived from the spinal cords of three rats each from the exercise and control groups 6 weeks after surgery. The clipped sections obtained from both ipsilateral and contralateral side were pooled. Isolated RNA (5 ng) was subjected to NGS library preparation using the SMARTer^®^ Stranded Total RNA Sample Prep Kit-Pico Input Mammalian (Clontech, TaKaRa). Each library (16 pM) was subjected to 2 × 75-bp paired-end sequencing sequenced on an Illumina MiSeq system using the MiSeq Reagent Kit v3-150 cycle (Illumina, San Diego, CA, USA). Bioinformatics analysis was performed using the following software: Tophat for gene mapping of the sequence data, Cufflinks to determine differences in expression levels, and DAVID Bioinformatics Resources 6.8 (National Institute of Allergy and Infectious Diseases (NIAID), NIH, Bethesda, MD, USA) for functional annotation of the gene list for coding mRNAs.

### 4.8. RT-PCR

Extracted total RNA was subjected to first-strand cDNA synthesis with random primers using the SuperScript^®^VILO cDNA Synthesis Kit (Thermo Fisher, Waltham, MA, USA). Products of reverse transcription were diluted 20-fold and used as templates for quantitative real-time PCR analysis (qRT-PCR) using SYBER Premix Ex Taq TMII (Tli RNase H Plus) on a 7500 Real Time PCR System (Applied Biosystems, Foster City, CA, USA), using cDNA derived from the exercise and control groups at 1, 3, and 6 weeks after surgery. Detected signals were confirmed as specific by a dissociation protocol. Data were normalized against the corresponding expression levels of *Gapdh*. Primer sets used for qRT-PCR were as follows:

*Rnf34* forward: CAGTCTGCTATGGTGCTGAGTT,
*Rnf34* reverse: TAGAGGTAGCACCCGCCTTCAT,
*Pacap* forward: CCTACCGCAAAGTCTTGGAC,
*Pacap* reverse: TTGACAGCCATTTGTTTTCG,
*Gapdh* forward: GAACATCATCCCTGAATCCA,
*Gapdh* reverse: CCAGTGAGCTTCCCGTTC.

### 4.9. Statistical Analysis

Wilcoxon signed-rank test was used to analyze pain reactions (pain thresholds) by mechanical stimulation. Student’s *t-*test was used for two-sample analyses to determine whether mRNA expression levels differed significantly between the exercise and control groups. Multi-way ANOVA (Analysis of Variance) was used for all of the RT-PCR data. Post-hoc Tukey HSD (Honestly Significant Difference) test was used to analyze the differences among the changes with time.

## 5. Conclusions

We demonstrated that pain relief in the SNL rats was achieved by 3 weeks of forced exercise, and that mRNA levels of Rnf34 and Pacap in the dorsal horn decreased relative to those in the no-exercise group. In this study, we employed next-generation sequencing in combination with laser microdissection methods in order to reveal the possible mechanism of exercise therapy in neuropathic pain, followed by the time course analysis of qPCR. To the best of our knowledge, such a comprehensive analysis of gene expression in dorsal horn where nociceptive information is processed is firstly reported here. We conclude that *Rnf34* and *Pacap* have been identified as potential candidates to imply direct and/or indirect correlation with pain-like behavior.

## Supplementary Materials

Supplementary materials can be found at www.mdpi.com/1422-0067/18/9/1962/s1.
