# Supplementary material for: Altered Gene Expression of RNF34 and PACAP Possibly Involved in Mechanism of Exercise-Induced Analgesia for Neuropathic Pain in Rats
Source: Int J Mol Sci. 2017 Sep 13;18(9):1962. doi: 10.3390/ijms18091962 (PMC5618611; doi:10.3390/ijms18091962)
Supplement: Supplementary file 1 [file ijms-18-01962-s001.pdf]

# Supplementary figure

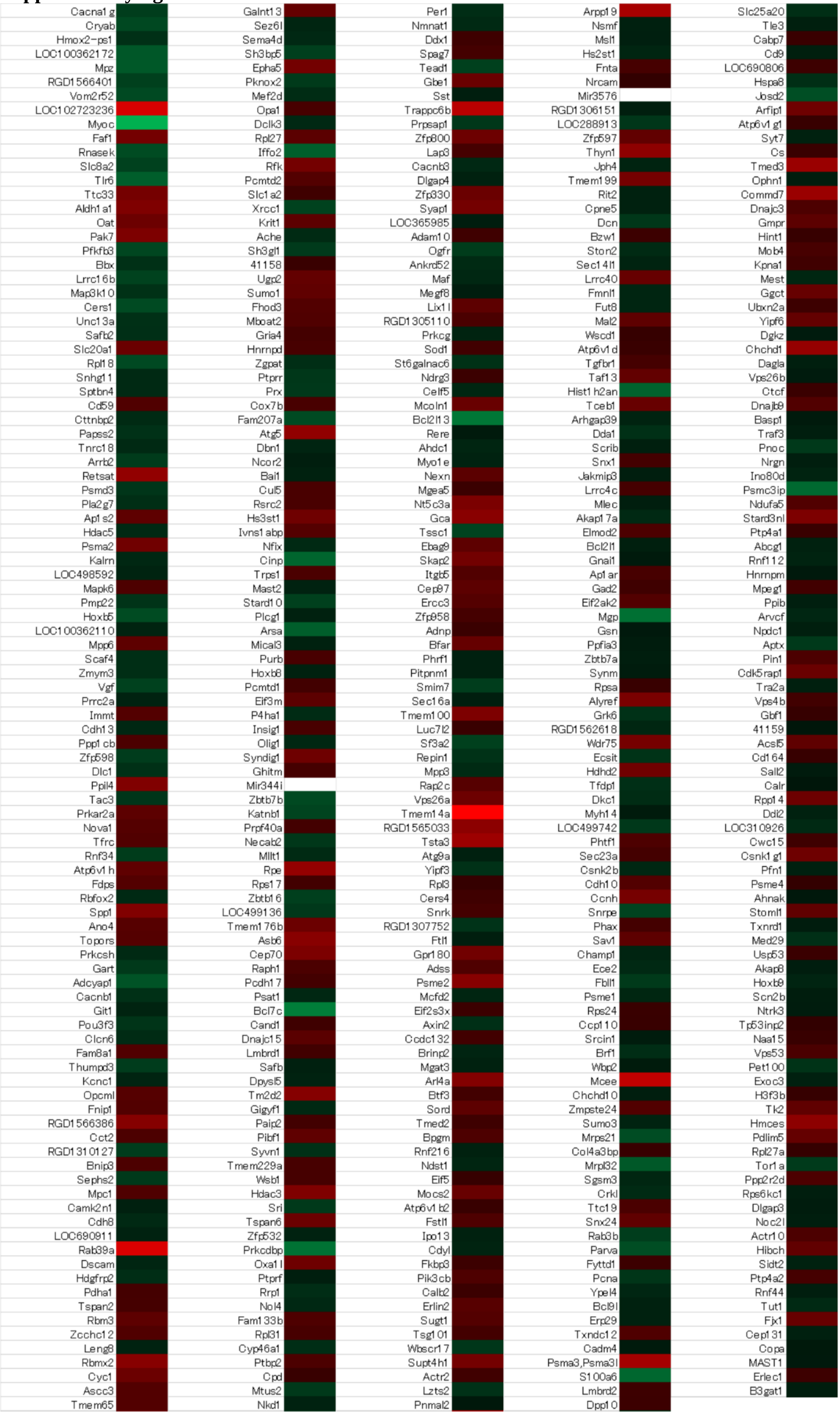

**Supplementary table**

**Basso, Beattie, and Bresnahan Locomotor Rating Scale**

| SCORE | Operating definitions of categories of attributes |
|---|---|
| 0 | No observable hindlimb (HL) movement |
| 1 | Slight movement of one or two joints, usually the hip and/or knee |
| 2 | Extensive movement of one joint or extensive movement of one joint and slight movement of one other joint |
| 3 | Extensive movement of two joints |
| 4 | Slight movement of all three joints of the HL |
| 5 | Slight movement of two joints and extensive movement of the third |
| 6 | Extensive movement of two joints and slight movement of the third |
| 7 | Extensive movement of all three joints of the HL |
| 8 | Sweeping with no weight support or plantar placement of the paw with no weight support |
| 9 | Plantar placement of the paw with weight support in stance only (i.e., when stationary) or occasional, frequent, or consistent weight-supported dorsal stepping and no plantar stepping |
| 10 | Occasional weight-supported plantar steps; no FL–HL coordination |
| 11 | Frequent to consistent weight-supported plantar steps and no FL–HL coordination |
| 12 | Frequent to consistent weight-supported plantar steps and occasional FL–HL coordination |
| 13 | Frequent to consistent weight-supported plantar steps and frequent FL–HL coordination |
| 14 | Consistent weight-supported plantar steps, consistent FL–HL coordination, and predominant paw position during locomotion is rotated (internally or externally) when it makes initial contact with the surface as well as just before it is lifted off at the end of stance; or frequent plantar stepping, consistent FL–HL coordination, and occasional dorsal stepping |
| 15 | Consistent plantar stepping and consistent FL–HL coordination and no toe clearance or occasional toe clearance during forward limb advancement; predominant paw position is parallel to the body at initial contact |
| 16 | Consistent plantar stepping and consistent FL–HL coordination during gait and toe clearance occurs frequently during forward limb advancement; predominant paw position is parallel at initial contact and rotated at lift off |
| 17 | Consistent plantar stepping and consistent FL–HL coordination during gait and toe clearance occurs frequently during forward limb advancement; predominant paw position is parallel at initial contact and lift off |
| 18 | Consistent plantar stepping and consistent FL–HL coordination during gait and toe clearance occurs consistently during forward limb advancement; predominant paw position is parallel at initial contact and rotated at lift off |
| 19 | Consistent plantar stepping and consistent FL–HL coordination during gait, toe clearance occurs consistently during forward limb advancement, predominant paw position is parallel at initial contact and lift off, and tail is down part or all of the time |
| 20 | Consistent plantar stepping and consistent coordinated gait, consistent toe clearance, predominant paw position is parallel at initial contact and lift off, and trunk instability; tail consistently up |
| 21 | Consistent plantar stepping and coordinated gait, consistent toe clearance, predominant paw position is parallel throughout stance, and consistent trunk stability; tail consistently up |

Originally published in Journal of Neurotrauma, Volume 12, Number 1, 1995.
